# Heat, Heatwaves and Cardiorespiratory Hospital Admissions in Helsinki, Finland

**DOI:** 10.3390/ijerph17217892

**Published:** 2020-10-28

**Authors:** Hasan Sohail, Virpi Kollanus, Pekka Tiittanen, Alexandra Schneider, Timo Lanki

**Affiliations:** 1 Department of Environmental and Biological Sciences, University of Eastern Finland, 70211 Kuopio, Finland; Timo.Lanki@thl.fi; 2 Department of Health Security, Finnish Institute for Health and Welfare (THL), 70701 Kuopio, Finland; virpi.kollanus@thl.fi (V.K.); pekka.tiittanen@thl.fi (P.T.); 3 Helmholtz Zentrum München-German Research Center for Environmental Health, Institute of Epidemiology, 85764 Neuherberg, Germany; alexandra.schneider@helmholtz-muenchen.de; 4 School of Medicine, University of Eastern Finland, 70211 Kuopio, Finland

**Keywords:** heat, heatwave, cardiovascular diseases, respiratory diseases, hospital admissions, climate change, ambient temperature, public health, time series, summer months

## Abstract

*Background:* There is a lack of knowledge concerning the effects of ambient heat exposure on morbidity in Northern Europe. Therefore, this study aimed to evaluate the relationships of daily summertime temperature and heatwaves with cardiorespiratory hospital admissions in the Helsinki metropolitan area, Finland. *Methods*: Time series models adjusted for potential confounders, such as air pollution, were used to investigate the associations of daily temperature and heatwaves with cause-specific cardiorespiratory hospital admissions during summer months of 2001–2017. Daily number of hospitalizations was obtained from the national hospital discharge register and weather information from the Finnish Meteorological Institute. *Results*: Increased daily temperature was associated with a decreased risk of total respiratory hospital admissions and asthma. Heatwave days were associated with 20.5% (95% CI: 6.9, 35.9) increased risk of pneumonia admissions and during long or intense heatwaves also with total respiratory admissions in the oldest age group (≥75 years). There were also suggestive positive associations between heatwave days and admissions due to myocardial infarction and cerebrovascular diseases. In contrast, risk of arrhythmia admissions decreased 20.8% (95% CI: 8.0, 31.8) during heatwaves. *Conclusions*: Heatwaves, rather than single hot days, are a health threat affecting morbidity even in a Northern climate.

## 1. Introduction

Global warming is anticipated to increase the frequency and intensity of hot days and heatwaves [1]. Exposure to high temperature can lead to many types of adverse physiological changes in the human body [2,3]. Therefore, evaluating the association of ambient heat exposure with human health is of paramount importance in a changing climate.

The positive association between ambient heat exposure and mortality has been well established in countries around the world [4,5,6,7,8]. Results from mortality studies have been the basis for the development of heat-health warning systems and action plans [9]. However, there has been less research on the association between heat exposure and hospital admissions. This is because hospital data is typically less easily accessible than mortality data. The majority of the studies on hospital admissions have included either all-cause admissions or the broad diagnosis categories of cardiovascular and respiratory diseases.

Interestingly, studies on heat exposure and hospital admissions have exhibited mixed results [10,11,12]. In addition to hospital admissions due to health conditions directly related to heat stress, fluid or electrolyte imbalance, and renal diseases [13,14,15], respiratory admissions are commonly found to be associated with heatwaves [16,17,18]. Associations have also been detected with hospital admissions due to some less studied disease outcomes [19,20,21,22]. Cardiovascular diseases have shown an association with hot days in some studies [11,12,23], but no such association was found in a meta-analysis by Turner et al. [24]. Hospital admission studies have focused on extreme heat leaving the need to investigate also the effects of moderate heat [10].

Polar amplification in the northern latitudes is making Northern Europe especially susceptible to the effects of global warming [25]. So far, only few studies have been carried out to evaluate the association of high ambient temperatures with hospital admissions in Northern European countries [17,26,27,28]. Therefore, this study investigates the association of heat and heatwaves with cardiorespiratory hospital admissions in the Helsinki metropolitan area in Finland. Understanding how morbidity, and not just mortality, is associated with heat exposure in Northern Europe is crucial for formulating adaptation strategies.

## 2. Methodology

### 2.1. Study Design and Population

Our time series study evaluated associations between daily mean temperature and daily number of non-elective hospital admissions in the Helsinki metropolitan area, Finland, in June—August 2001–2017. The Helsinki metropolitan area includes the cities of Helsinki, Vantaa, Espoo and Kauniainen. The average population size of the area was approximately one million during the period of 2001–2017 [29].

### 2.2. Health and Environmental Data

Health data was obtained from a national registry containing information on daily hospital admissions. The study contained information on daily non-elective hospital admission in June–August 2001–2017 for all cardiovascular diseases (CVD: I00–I99), all respiratory diseases (J00–J99), myocardial infarction (MI: I21–I22), ischemic heart disease (IHD: I20–I25), cerebrovascular diseases (I60–I61, I63–I64), arrhythmia (I46.0, I46.9, I47–I49), asthma (J45, J46), chronic obstructive pulmonary disease (COPD: J41, J44), and pneumonia (J12–J15, J16.8, J18) in specific age groups (all ages, 18–64, 65–74 and ≥75).

Exposures of interest in the study were daily mean temperature and heatwaves. Daily weather data was provided by the Finnish Meteorological Institute. Data was collected using a fixed weather station at the Helsinki-Vantaa airport. Air pollution data was obtained from the Helsinki Region Environmental Services Authority, and included information on nitrogen dioxide (NO_2_), ozone (O_3_), inhalable particulate matter (PM_10_; aerodynamic diameter ≤ 10 μm) and fine particulate matter (PM_2.5_; aerodynamic diameter ≤ 2.5 μm). For NO_2,_ PM_10_ and PM_2.5_, Kallio urban background station was used. Daily averages were calculated from 1-h values and at least 18 hourly values had to be available, otherwise daily average was defined as missing. For ozone (O_3_), 8-h moving averages were calculated using hourly data from Luukki measurement site, which is an aerial background station. First, at least 6 hourly values had to be available; otherwise the 8-h moving average was defined as missing. Second, maximum daily 8-h moving averages were calculated from 8-h moving averages and at least 18 values had to be available, otherwise the maximum 8-h moving average was defined as missing. Data on daily pollen counts was provided by the University of Turku Aerobiology Unit.

### 2.3. Statistical Analysis

Multivariate Poisson time series regression models were used to evaluate whether daily number of hospital admissions is associated with: (1) daily mean temperature or (2) heatwaves. Previous studies have reported that the effect of high temperature on hospital admissions takes place only with a short delay. Therefore, in the analyses on the effects of daily mean temperature we looked at individual daily lags from lag 0 (exposure during the day of admission) to lag 5 (exposure 5 days prior to the day of admission). We used the average of lags 0 and 1 as the main exposure variable.

In the second set of analyses, we investigated the effect of heatwaves (extended heat exposure) by adding an indicator variable for heatwaves in the model (without daily temperature). All days belonging to heatwaves got a value of 1, all other days were 0. There is no standard definition for a heatwave, but varying combinations of intensity and duration have been used in earlier studies. In the current study, heatwaves were defined using 90th and 95th percentile cutoff points for mean daily temperature in May–August 2001–2017. For heatwaves that lasted nine days or less, the cutoff temperature had to be exceeded in each consecutive day. For heatwaves that lasted ten days or longer, one day with temperature below the cutoff point after the tenth day was allowed, if the cutoff temperature was then again exceeded for at least two consecutive days. At the 90th percentile cutoff point, heatwaves were defined as periods lasting for four or more days, and analyses were also conducted separately for short heatwaves (4–7 days) and long heatwaves (10 or more days). At the 95th cutoff point, length of heatwave was 3 or more days.

Time trends were modelled with a three-way interaction between year, month and day of the week and an indicator for holidays. Linear terms for air pollutants (NO_2,_ O_3_, PM_10_ and PM_2.5_), relative humidity (RH), and barometric pressure (BP) were introduced as potential confounders (selected a priori) in the model. Both lag 0 and the average of lags 1–3 were used for air pollutants, relative humidity, and barometric pressure. Moreover, pollen count (a sum of alder, birch, mug wort, and grasses) was taken into account using two indicators. The daily count (lag 0) and the sum of count on three previous days (lags 1–3) were categorized into two categories using 100 grains/m^3^ as a cutoff.

Poisson regression was applied using the glm function in the stats package in R (R Development Core Team, Vienna, Austria) [30]. Overdispersion was checked for using the R package AER, but no evidence of that was found. Effect estimates were reported as percentage change with 95% confidence intervals. We performed analysis separately for all ages and age groups 18–64, 65–74 and ≥75 years. The shape of association of continuous exposure and covariates were checked with the gam function in the mgcv package using thin plate regression splines. This technique has advantage of not requiring the limitation of choosing the knot locations. No evidence of non-linearity was found for daily temperature; in the supplement are presented two examples of exposure-response functions (Supplemental Figures S1 and S2).

As a sensitivity analysis, we ran separate models for daily average temperature by removing days with low temperature (1st percentile, 9.4 °C) and days with high temperature (99th percentile, 24.5 °C). The models were also run without air pollutant variables to check the robustness of our results.

## 3. Results

Table 1 and Table 2 show descriptive statistics for the outcome and exposure variables used in this study. The majority (46.3%) of all cardiorespiratory hospital admissions occurred among persons aged ≥75 years. The mean daily temperature during the study period was 16.8 °C. The average daily mean humidity was 72%. Daily mean concentrations of PM_2.5_, PM_10_, O_3_ and NO_2_ were 7.6, 13.2, 76.8, and 17.0 µg/m^3^, respectively.

Table 3 shows the number of heatwave days and mean daily temperature during heatwave and non-heatwave days.

Table 4 shows the associations of daily temperature, average of lags 0 and 1, with hospital admissions due to cardiorespiratory diseases. No statistically significant increased risk was found for any of the disease categories. Similar results were obtained in the analyses on individual daily lags (Supplemental File S1–S6). There was a suggestive positive association for arrhythmia admissions in the 65–74 age group. Respiratory diseases showed a statistically significant protective association among all ages, while asthma showed a significant protective association in all ages and the 65–74 age group. All cardiovascular diseases and COPD showed borderline significant protective associations in the 18–64 and ≥75 age groups, respectively.

Associations between heatwaves, i.e., heatwave days, and cardiorespiratory hospital admissions varied depending on the type of disease (Table 5). In the analysis where 90th percentile of the temperature distribution was used as a cutoff point, statistically significant positive associations were detected between all heatwaves and pneumonia in all ages and the 18–64 age group and all respiratory diseases in the 18–64 age group. We also found suggestive increased risk for cerebrovascular disease in all ages and COPD hospitalizations in the 18–64 age group. Protective effects were detected for arrhythmia. When short heatwaves were analyzed separately, we found significant positive associations also with COPD admissions in the 18–64 age group and MI in the 65–74 age group.

Long heatwaves (90th percentile cutoff point) and more intense heatwaves (95th percentile) showed a statistically significant increased risk for all respiratory hospitalizations in the age group ≥ 75. For hospital admissions due to pneumonia, there was a statistically significant positive association with long heatwaves and a suggestive positive association with intense heatwaves among those aged ≥ 75. We evaluated the robustness of our results by performing sensitivity analyses. Results remained essentially the same (the results are not shown).

## 4. Discussion

This study investigated separately the associations of daily mean temperature and heatwaves with hospital admissions for cardiorespiratory diseases in summer months (June–August) in the Helsinki metropolitan area, Finland. We found that daily mean temperature was associated with a decreased risk of hospitalization for all respiratory diseases, asthma, and to a lesser extent cardiovascular diseases. In contrast, during heatwaves the risk of hospitalization increased due to pneumonia, and in some age groups for respiratory diseases in general, COPD and MI. We also found a negative association between heatwaves and arrhythmia hospitalizations.

Our study results showed no increased risk for all cardiovascular admissions in association with daily mean temperature or heatwaves. These findings are in line with a review, in which Turner et al. found no association between summertime temperature and cardiovascular morbidity [24]. Likewise, many later studies have shown either no or weak association between heat and cardiovascular morbidity [16,31,32,33]. One potential reason could be that most of the vulnerable people suffering acute cardiovascular events die before reaching hospital, which may explain the lack of effect on cardiovascular hospital admissions [17].

Our results suggest that heatwaves may yet be associated with specific subtypes of cardiovascular diseases. We found a borderline significant association between heatwave days and cerebrovascular admissions among all ages. However, there has been no evidence in previous literature for an effect of heat or heatwaves on cerebrovascular admissions, and no association with cerebrovascular morbidity was found in an overview of review studies [34]. Our study also indicates a positive association between heatwaves and myocardial infarction admissions in the 65–74 age group, which is in contrast with previous studies that found no or a decreased risk of MI admissions during summer months [27,35,36].

We also observed a protective association between heatwaves and hospital admissions due to arrhythmia. In contrast, a population based study from Canada did not find any association between heat and arrhythmia admissions [37]. So far, there are too few studies to draw conclusions on the effect on arrhythmia.

We found a positive association of heatwaves with hospital admissions due to respiratory diseases in general, pneumonia and COPD. These results are consistent with the findings from previous studies on respiratory morbidity [16,17,18,38,39]. A possible explanation for a stronger association between heatwaves and respiratory admissions than between heatwaves and cardiovascular admissions is that respiratory diseases are not as acutely fatal as cardiovascular diseases. This means that more people are able to reach hospitals. During heat exposure human body maintains safe body temperature through thermoregulation, which results in an increase in skin blood flow, cardiac output, and pulmonary ventilation [40,41,42]. Hyperthermia can also lead to thermal hyperpnea (increase in respiratory rate and tidal volume) [40]. These responses can lead to exacerbation of respiratory diseases and an increase in hospitalizations. Moreover, it has been suggested that acute respiratory effects can be caused by the direct effect of breathing hot air [18].

In our study, long heatwaves and more intense heatwaves (95th percentile cutoff point) were associated with an increased risk of total respiratory and pneumonia hospitalizations in the oldest age category. These findings are in line with other studies, which have reported elderly people as the most vulnerable group for respiratory morbidity effects due to high daily temperatures and heatwaves [17,31,43,44]. However, with different definitions of heatwaves we also found associations between heatwaves and respiratory health in the age group 18–64.

The risk of morbidity associated with heatwaves may vary depending on the length and intensity of the hot period. In a study by Levy et al., sustained high heat index increased the risk of emergency department arrivals [45]. Likewise, a study in Australia found a higher risk of infants’ hospitalizations with longer duration heatwaves [46]. The effect of heatwaves on morbidity may vary across different regions and still needs more investigation.

Finland, being a North European country, has cool summer temperatures. This is most likely the reason that we found no positive associations or even some protective associations in connection with daily temperature. However, our results do suggest that heatwaves pose a public health concern regarding morbidity (as indicated by hospital admissions).

One of the strengths of our study is that it was conducted in a Northern climate where only few studies have taken place. We used long time series data and had in practice a hundred percent coverage of hospital admissions. Thus, reliable evidence on population level associations could be provided. Also, this study evaluated the associations of temperature with cause-specific diagnosis and in age groups rather than for broader disease categories and total population. The potential confounding effect of air pollution was controlled using all major indicators of air pollution. However, this study has some limitations as well. We were not able to include persons younger than 18 years because the limited number of cases in the age group would have led to imprecise effect estimates. Further, the number of events was low also in some age-stratified analyses on sub-diagnoses, e.g., on asthma, which has to be taken into account when interpreting the results. Finally, the study only included cardiovascular and respiratory hospital admissions. Admissions due to other causes could also be affected by heat exposure.

## 5. Conclusions

In conclusion, we found no associations and even protective associations between daily mean temperature and cardiorespiratory hospital admissions. However, our results do suggest that heatwaves are a health threat and affect morbidity even in the northern climate. Moreover, it is not only the elderly who are at risk. Preventive measures should also take other age groups into account.

## Figures and Tables

**Table 1:** Daily number of hospital admissions for cardiorespiratory diseases during summer months (June–August) in the Helsinki metropolitan area, Finland, 2001–2017.

Disease	All Ages			Age 18–64			Age 65–74			Age ≥ 75		
Cardiovascular	Median	Min.	Max.	Median	Min.	Max.	Median	Min.	Max.	Median	Min.	Max.
All	24	6	45	6	0	19	5	0	19	13	2	31
MI ^1^	3	0	11	1	0	5	0	0	6	1	0	9
IHD ^2^	5	0	20	1	0	8	1	0	9	2	0	12
Cerebrovascular	4	0	12	1	0	7	1	0	7	2	0	10
Arrhythmia	4	0	14	1	0	5	1	0	7	2	0	10
**Respiratory**												
All	16	2	37	5	0	20	3	0	12	3	0	12
Asthma	1	0	8	0	0	4	0	0	3	0	0	3
COPD ^3^	2	0	9	0	0	4	1	0	6	1	0	7
Pneumonia	5	0	23	1	0	11	1	0	7	2	0	15

^1^ Myocardial Infarction, ^2^ Ischemic Heart Disease, ^3^ Chronic Obstructive Pulmonary Disease.

**Table 2:** Daily levels of temperature, relative humidity, barometric pressure, and air pollutants during summer months (June–August) in the Helsinki metropolitan area, Finland, 2001–2017.

	Mean	Median	Min.	Max.	SD.	Missing Values
**Meteorology**						
Temperature [°C]	16.8	16.7	6.1	26.6	3.4	0
Relative Humidity [%]	72	73	37	98	12.4	0
Barometric Pressure [hPa]	1012	1012	986	1034	7.1	0
**Air pollution** [µg/m^3^]						
Ozone	76.8	75.9	19.4	156.0	17.6	114
PM_2.5_	7.6	6.6	0.5	50.2	4.6	32
PM_10_	13.2	11.9	1.6	62.9	6.5	24
NO_2_	17.0	16.0	2.9	62.6	7.5	23

**Table 3:** Number of heatwave days and mean temperature during heatwave and non-heatwave days during summer months (June–August) in the Helsinki metropolitan area, Finland, 2001–2017.

	Number of Heatwave Days	Average Temperature During Heatwaves [°C]	Average Temperature During Control Days [°C]
**90th percentile**			
All heatwaves	113	22.8	16.3
Short heatwaves	62	22.6	16.3
Long heatwaves	51	23.1	16.3
**95th percentile**			
All Heatwaves	60	23.9	16.6

**Table 4:** Percentage change (95% CI) in daily hospital admissions for cardiorespiratory diseases associated with a 1 °C increase in daily mean temperature during summer months (June–August) in the Helsinki metropolitan area, Finland, 2001–2017.

Disease	All Ages	Age 18–64	Age 65–74	Age ≥ 75
**Cardiovascular**				
All	−0.49 (−1.14, 0.17)	−1.21 (−2.47, 0.07)	0.02 (−1.38, 1.44)	−0.32 (−1.22, 0.60)
MI ^1^	0.14 (−1.70, 2.02)	−0.17 (−3.50, 3.28)	0.36 (−3.52, 4.39)	0.16 (−2.51, 2.90)
IHD ^2^	−0.46 (−1.79, 0.88)	−0.73 (−3.22, 1.82)	−0.40 (−3.14, 2.42)	−0.37 (−2.26, 1.57)
Cerebrovascular	−0.70 (−2.22, 0.85)	−0.89 (−3.78, 2.09)	−1.37 (−4.44, 1.81)	−0.19 (−2.38, 2.06)
Arrhythmia	−0.66 (−2.24, 0.95)	−2.44 (−5.58, 0.80)	2.95 (−0.48, 6.50)	−1.52 (−3.69, 0.69)
**Respiratory**				
All	−1.09 (−1.88, −0.30)	0.53 (−0.90, 1.97)	−0.59 (−2.43, 1.28)	−0.11 (−1.45, 1.25)
Asthma	−3.49 (−6.25, −0.65)	3.25 (−2.11, 8.91)	−8.96 (−16.42, −0.82)	−2.68 (−8.06, 3.01)
COPD ^3^	−0.53 (−2.56, 1.55)	1.54 (−2.81, 6.09)	0.97 (−2.60, 4.67)	−2.59 (−5.57, 0.49)
Pneumonia	−0.30 (−1.67, 1.09)	−0.66 (−3.17, 1.92)	−0.33 (−3.47, 2.92)	1.12 (−0.94, 3.22)

^1^ Myocardial Infarction, ^2^ Ischemic Heart Disease, ^3^ Chronic Obstructive Pulmonary Disease.

**Table 5:** Percentage change (95% CI) in daily hospital admissions for cardiorespiratory diseases associated with heatwave days in summer months (June–August) in the Helsinki metropolitan area, Finland, 2001–2017.

Heatwaves	Disease	All Ages	Age 18–64	Age 65–74	Age ≥ 75
90th percentile cutoff point, all heatwaves	**Cardiovascular**				
	All	−2.14 (−7.71, 3.76)	−7.57 (−17.73, 3.84)	2.01 (−10.00, 15.62)	−0.57 (−8.30, 7.81)
	MI ^1^	4.77 (−11.03, 23.38)	−6.82 (−31.64, 27.00)	40.42 (−0.43, 98.04)	−3.55 (−23.81, 22.09)
	IHD ^2^	0.06 (−11.24, 12.79)	−3.50 (−23.35, 21.49)	9.62 (−14.50, 40.55)	−2.81 (−18.03, 15.23)
	Cerebrovascular	12.17 (−1.93, 28.29)	12.12 (−14.10, 46.35)	17.96 (−10.56, 55.57)	10.07 (−8.85, 32.93)
	Arrhythmia	−20.78 (−31.81, −7.97)	−34.41 (−52.71, −9.03)	−13.13 (−35.68, 17.31)	−18.44 (−33.63, 0.24)
	**Respiratory**				
	All	4.18 (−3.04, 11.94)	17.97 (3.97, 33.86)	−6.11 (−20.27, 10.57)	10.18 (−2.05, 23.94)
	Asthma	−4.36 (−27.46, 26.08)	17.86 (−28.57, 94.46)	−33.27 (−68.73, 42.42)	38.56 (−15.15, 126.25)
	COPD ^3^	−5.03 (−20.95, 14.10)	48.05 (0.00, 119.20)	−9.48 (−33.48, 23.18)	−20.67 (−40.38, 5.55)
	Pneumonia	20.48 (6.85, 35.85)	28.94 (3.00, 61.42)	25.67 (−4.24, 64.93)	18.25 (−0.82, 40.98)
90th percentile cutoff point, short heatwaves	**Cardiovascular**				
	All	−1.32 (−8.05, 5.91)	−3.64 (−16.10, 10.68)	3.48 (−11.15, 20.53)	−1.76 (−10.92, 8.35)
	MI ^1^	17.13 (−3.44, 42.07)	5.21 (−28.12, 54.01)	74.86 (15.75, 164.16)	2.36 (−21.99, 34.31)
	IHD ^2^	8.47 (−5.70, 24.78)	2.63 (−22.04, 35.11)	21.29 (−9.64, 62.81)	5.47 (−13.30, 28.31)
	Cerebrovascular	12.27 (−4.19, 31.56)	19.07 (−12.40, 61.84)	4.48 (−25.99, 47.49)	12.23 (−9.94, 39.87)
	Arrhythmia	−26.85 (−39.36, −11.76)	−33.82 (−55.37, −1.87)	−10.75 (−38.04, 28.56)	−32.07 (−48.07, −11.15)
	**Respiratory**				
	All	5.24 (−3.58, 14.86)	27.03 (9.49, 47.38)	−3.89 (−21.44, 17.59)	−1.04 (−14.72, 14.85)
	Asthma	21.58 (−10.78, 65.68)	47.11 (−18,23, 164.64)	0.23 (−57.34, 135.48)	72.41 (−1.01, 200.29)
	COPD ^3^	−6.98 (−25.78, 16.58)	65.33 (3.13, 165.06)	−10.06 (−38.48, 31.50)	−28.29 (−49.87, 2.59)
	Pneumonia	20.31 (3.49, 39.87)	48.30 (13.87, 93.15)	33.99 (−4.80, 88.59)	−0.30 (−21.09, 25.96)
90th percentile cutoff point, long heatwaves	**Cardiovascular**				
	All	−3.01 (−11.46, 6.24)	−13.56 (−28.14, 3.97)	−1.46 (−18.64, 19.35)	2.50 (−9.60, 16.23)
	MI ^1^	−12.85 (−32.96, 13.31)	−18.09 (−49.11, 31.86)	−6.02 (−44.48, 59.11)	−12.16 (−40.86, 30.47)
	IHD ^2^	−13.36 (−28.82, 5.47)	−11.50 (−38.68, 27.73)	−6.97 (−37.40, 38.24)	−17.85 (−38.41, 9.58)
	Cerebrovascular	7.87 (−13.15, 33.98)	−12.16 (−43.75, 37.16)	38.92 (−7.90, 109.53)	4.53 (−23.67, 43.14)
	Arrhythmia	−8.34 (−26.27, 14.57)	−29.16 (−57.62, 18.40)	−24.17 (−52.31, 20.57)	10.09 (−18.10, 47.98)
	**Respiratory**				
	All	−1.01 (−11.29, 10.46)	−0.85 (−18.99, 21.35)	−12.54 (−31.42, 11.54)	22.47 (3.14, 45.43)
	Asthma	−52.46 (−71.80, 19.74)	−22.69 (−67.96, 86.48)	−76.19 (−93.91, −6.86)	−25.76 (−67.86, 71.52)
	COPD ^3^	−1.63 (−25.35, 29.63)	35.11 (−26.14, 147.14)	−7.93 (−42.00, 46.17)	−10.78 (−41.60, 36.30)
	Pneumonia	18.20 (−0.94, 41.03)	−0.82 (−31.20, 42.97)	11.85 (−24.42, 65.53)	40.65 (9.87, 80.05)
95th percentile cutoff point, all heatwaves	**Cardiovascular**				
	All	−2.43 (−9.60, 5.31)	−12.15 (−24.15, 2.23)	−1.07 (−15.82, 16.25)	2.22(−8.07, 13.67)
	MI ^1^	3.02 (−17.04, 27.93)	−13.29 (−43.04, 32.00)	9.38 (−29.32, 69.27)	8.03 (−21.07, 47.85)
	IHD ^2^	1.83 (−13.03, 19.24)	−14.20 (−37.31, 17.42)	3.11 (−24.99, 41.72)	9.44 (−12.54, 36.95)
	Cerebrovascular	−3.86 (−19.65, 15.05)	−34.11 (−54.61, −4.36)	13.26 (−20.43, 61.20)	6.23 (−17.64, 37.00)
	Arrhythmia	−14.67 (−29.62, 3.45)	−13.72 (−41.67, 27.61)	−18.75 (−45.62, 21.41)	−13.82 (−34.07, 12.66)
	**Respiratory**				
	All	1.77 (−7.27, 11.71)	−0.37 (−15.56, 17.57)	−18.93 (−34.51, 0.36)	22.46 (5.62, 41.98)
	Asthma	11.30 (−24.55, 64.21)	27.09 (−38.56, 162.86)	−28.62 (−72.94, 88.25)	68.89 (−8.60, 212.10)
	COPD ^3^	4.93 (−16.87, 32.44)	42.26 (−15.37, 139.15)	−25.69 (−50.41, 11.37)	18.38 (−16.41, 67.63)
	Pneumonia	6.80 (−8,66, 24.88)	−5.95 (−30.44, 27.17)	3.51 (−27.49, 47.76)	24.16 (−0.64, 55.15)

^1^ Myocardial Infarction, ^2^ Ischemic Heart Disease, ^3^ Chronic Obstructive Pulmonary Disease.

## Supplementary Materials

The following are available online at https://www.mdpi.com/1660-4601/17/21/7892/s1, Figure S1: Shape of association between daily mean temperature and cardiovascular hospital admissions during summer months in Helsinki metropolitan area, Finland, 2001–2017, Figure S2: Shape of association between daily mean temperature and respiratory admissions during summer months in Helsinki, metropolitan area, Finland, 2001–2017, File S1: Lag 0; File S2: Lag 1; File S3: Lag 2; File S4: Lag 3; File S5: Lag 4; File S6: Lag 5.
