# Supplementary material for: Heat, Heatwaves and Cardiorespiratory Hospital Admissions in Helsinki, Finland
Source: Int J Environ Res Public Health. 2020 Oct 28;17(21):7892. doi: 10.3390/ijerph17217892 (PMC7663418; doi:10.3390/ijerph17217892)
Supplement: Supplementary file 1 [file ijerph-17-07892-s001.pdf]

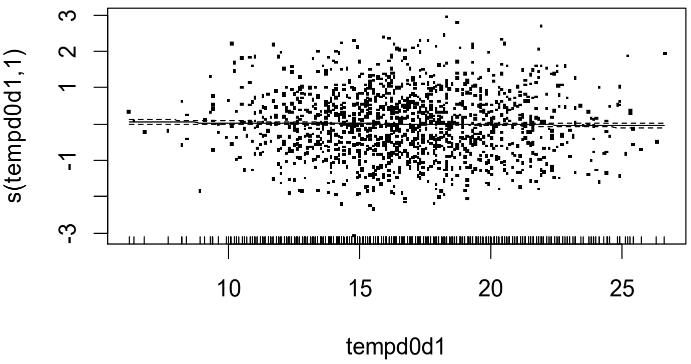

Figure S1. Shape of association between daily mean temperature and cardiovascular hospital admissions during summer months in Helsinki metropolitan area, Finland, 2001–2017.

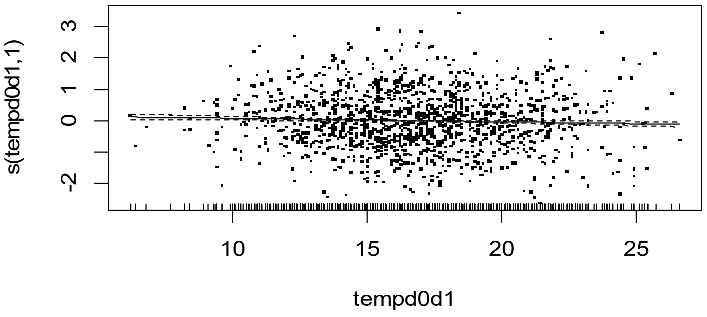

Figure S2. Shape of association between daily mean temperature and respiratory admissions during summer months in Helsinki metropolitan area, Finland, 2001–2017.

File S1: Lag 0

Table 1. Percentage change in daily hospital admissions for cardiorespiratory diseases (All-ages) associated with a 1 °C increase in daily mean temperature during summer months in the Helsinki metropolitan area, Finland, 2001–2017.

Outcome	% change RR	Low CI (%)	Up CI(%)	*p*-value
Cardiovascular	−0.52	−0.95	−0.08	0.02
Respiratory	−1.47	−1.97	−0.98	0.00
MI	−0.35	−1.59	0.91	0.59
IHD	−0.37	−1.29	0.55	0.42
Cerebrovascular	−0.22	−1.25	0.82	0.68
Arrhythmia	−1.33	−2.37	−0.28	0.01
Asthma	−2.66	−4.39	−0.89	0.00
COPD	−0.04	−1.40	1.33	0.95
Pneumonia	−0.98	−1.84	−0.12	0.03

Table 2. Percentage change in daily hospital admissions for cardiorespiratory diseases (18–64 years age group) associated with 1 °C increase in daily mean temperature during summer months in Helsinki metropolitan area, Finland, 2001–2017.

Outcome	% change RR	Low CI (%)	Up CI(%)	*p*-value
Cardiovascular	−1.03	−1.88	−0.17	0.02
Respiratory	−0.78	−1.70	0.16	0.10
MI	−0.66	−2.89	1.62	0.57
IHD	−0.52	−2.23	1.21	0.55
Cerebrovascular	−0.51	−2.50	1.53	0.62
Arrhythmia	−1.55	−3.65	0.59	0.15
Asthma	−1.02	−4.32	2.39	0.55
COPD	1.59	−1.29	4.56	0.28
Pneumonia	−1.39	−3.01	0.25	0.10

Table 3. Percentage change in daily hospital admissions for cardiorespiratory diseases (65–74 years age group) associated with 1 °C increase in daily mean temperature during summer months in Helsinki metropolitan area, Finland, 2001–2017.

Outcome	% change RR	Low CI (%)	Up CI(%)	*p*-value
Cardiovascular	−0.25	−1.19	0.69	0.60
Respiratory	−0.84	−2.02	0.36	0.17
MI	−0.56	−3.16	2.11	0.68
IHD	−0.33	−2.21	1.58	0.73
Cerebrovascular	0.32	−1.73	2.40	0.76
Arrhythmia	0.79	−1.45	3.08	0.49
Asthma	−3.83	−8.87	1.49	0.15
COPD	−0.71	−3.01	1.64	0.55
Pneumonia	−1.57	−3.52	0.43	0.12

Table 4. Percentage change in daily hospital admissions for cardiorespiratory diseases (≥75 years age group) associated with 1 °C increase in daily mean temperature during summer months in Helsinki metropolitan area, Finland, 2001–2017.

Outcome	% change RR	Low CI (%)	Up CI(%)	*p*-value
Cardiovascular	−0.39	−1.07	0.22	0.21
Respiratory	−0.66	−1.72	0.21	0.13
MI	−0.02	−0.97	1.83	0.98
IHD	−0.29	−1.25	1.04	0.66
Cerebrovascular	−0.34	−1.41	1.16	0.66
Arrhythmia	−2.28	−5.12	−0.83	0.00
Asthma	−1.50	−4.67	2.06	0.40
COPD	−0.32	−1.68	1.78	0.76
Pneumonia	0.08	−0.51	1.40	0.91

File S2: Lag 1

Table 1. Percentage change in daily hospital admissions for cardiorespiratory diseases (All-ages) associated with 1 °C increase in daily mean temperature during summer months in Helsinki metropolitan area, Finland, 2001–2017.

Outcome	% change	Low CI (%)	Up CI(%)	*p*-value
Cardiovascular	−0.31	−0.69	0.07	0.11
Respiratory	−0.85	−1.28	−0.41	0.00
MI	0.04	−1.05	1.14	0.95
IHD	0.00	−0.80	0.81	1.00
Cerebrovascular	−0.34	−1.23	0.57	0.46
Arrhythmia	−0.58	−1.49	0.34	0.22
Asthma	−1.95	−3.49	−0.38	0.01
COPD	0.42	−0.76	1.61	0.49
Pneumonia	−0.41	−1.16	0.35	0.29

Table 2. Percentage change in daily hospital admissions for cardiorespiratory diseases (18–64 years age group) associated with 1 °C increase in daily mean temperature during summer months in Helsinki metropolitan area, Finland, 2001–2017.

Outcome	% change	Low CI (%)	Up CI(%)	*p*-value
Cardiovascular	−0.40	−1.14	0.36	0.30
Respiratory	−0.34	−1.15	0.48	0.42
MI	−0.62	−2.56	1.36	0.54
IHD	−0.42	−1.91	1.10	0.59
Cerebrovascular	0.36	−1.39	2.13	0.69
Arrhythmia	−0.91	−2.76	0.97	0.34
Asthma	0.62	−2.31	3.63	0.68
COPD	2.18	−0.36	4.78	0.09
Pneumonia	−1.54	−2.96	−0.11	0.04

Table 3. Percentage change in daily hospital admissions for cardiorespiratory diseases (65–74 years age group) associated with 1 °C increase in daily mean temperature during summer months in Helsinki metropolitan area, Finland, 2001–2017.

Outcome	% change	Low CI (%)	Up CI(%)	*p*-value
Cardiovascular	−0.17	−0.99	0.65	0.68
Respiratory	−0.19	−1.23	0.85	0.72
MI	0.84	−1.44	3.18	0.47
IHD	0.32	−1.32	2.00	0.70
Cerebrovascular	−0.51	−2.28	1.30	0.58
Arrhythmia	0.91	−1.04	2.89	0.36
Asthma	−4.07	−8.48	0.55	0.08
COPD	0.46	−1.57	2.53	0.66
Pneumonia	−0.94	−2.66	0.81	0.29

Table 4. Percentage change in daily hospital admissions for cardiorespiratory diseases (≥75 years age group) associated with 1 °C increase in daily mean temperature during summer months in Helsinki metropolitan area, Finland, 2001–2017.

Outcome	% change	Low CI (%)	Up CI(%)	*p*-value
Cardiovascular	−0.34	−0.86	0.20	0.22
Respiratory	−0.18	−0.92	0.58	0.65
MI	0.11	−1.49	1.74	0.89
IHD	0.09	−1.06	1.25	0.88
Cerebrovascular	−0.56	−1.84	0.74	0.40
Arrhythmia	−1.13	−2.39	0.14	0.08
Asthma	−1.67	−4.71	1.46	0.29
COPD	−0.50	−2.26	1.30	0.58
Pneumonia	0.70	−0.44	1.84	0.23

File S3: Lag 2

Table 1. Percentage change in daily hospital admissions for cardiorespiratory diseases (All-ages) associated with 1 °C increase in daily mean temperature during summer months in Helsinki metropolitan area, Finland, 2001–2017.

Outcome	% change	Low CI (%)	Up CI(%)	*p*-value
Cardiovascular	−0.47	−0.81	−0.12	0.01
Respiratory	−0.63	−1.03	−0.23	0.00
MI	−0.27	−1.27	0.74	0.59
IHD	−0.35	−1.08	0.38	0.35
Cerebrovascular	−0.49	−1.30	0.33	0.24
Arrhythmia	−0.81	−1.65	0.03	0.06
Asthma	−1.08	−2.51	0.37	0.14
COPD	0.29	−0.80	1.38	0.60
Pneumonia	0.00	−0.69	0.70	0.99

Table 2. Percentage change in daily hospital admissions for cardiorespiratory diseases (18–64 years age group) associated with 1 °C increase in daily mean temperature during summer months in Helsinki metropolitan area, Finland, 2001–2017.

Outcome	% change	Low CI (%)	Up CI(%)	*p*-value
Cardiovascular	−0.66	−1.33	0.03	0.06
Respiratory	−0.17	−0.92	0.58	0.65
MI	−1.14	−2.91	0.65	0.21
IHD	−0.87	−2.22	0.50	0.21
Cerebrovascular	0.11	−1.46	1.70	0.90
Arrhythmia	−0.96	−2.66	0.77	0.27
Asthma	1.41	−1.25	4.15	0.30
COPD	1.39	−0.88	3.71	0.23
Pneumonia	−0.58	−1.91	0.76	0.39

Table 3. Percentage change in daily hospital admissions for cardiorespiratory diseases (65–74 years age group) associated with 1°C increase in daily mean temperature during summer months in Helsinki metropolitan area, Finland, 2001–2017.

Outcome	% change	Low CI (%)	Up CI(%)	*p*-value
Cardiovascular	−0.04	−0.79	0.72	0.92
Respiratory	−0.17	−1.12	0.79	0.73
MI	0.51	−1.60	2.67	0.64
IHD	0.02	−1.48	1.54	0.98
Cerebrovascular	−0.26	−1.89	1.40	0.75
Arrhythmia	0.44	−1.35	2.27	0.63
Asthma	−1.38	−5.46	2.88	0.52
COPD	0.38	−1.48	2.28	0.69
Pneumonia	−0.65	−2.25	0.97	0.43

Table 4. Percentage change in daily hospital admissions for cardiorespiratory diseases (≥75 years age group) associated with 1 °C increase in daily mean temperature during summer months in Helsinki metropolitan area, Finland, 2001–2017.

Outcome	% change	Low CI (%)	Up CI(%)	*p*-value
Cardiovascular	−0.56	−1.04	−0.07	0.02
Respiratory	0.08	−0.60	0.77	0.82
MI	−0.08	−1.55	1.42	0.92
IHD	−0.23	−1.28	0.84	0.67
Cerebrovascular	−0.92	−2.10	0.27	0.13
Arrhythmia	−1.35	−2.49	−0.19	0.02
Asthma	−0.93	−3.75	1.98	0.53
COPD	−0.44	−2.08	1.23	0.61
Pneumonia	0.78	−0.24	1.82	0.13

File S4: Lag 3

Table 1. Percentage change in daily hospital admissions for cardiorespiratory diseases (All-ages) associated with 1 °C increase in daily mean temperature during summer months in Helsinki metropolitan area, Finland, 2001–2017.

Outcome	% change	Low CI (%)	Up CI(%)	*p*-value
Cardiovascular	−0.47	−0.81	−0.12	0.01
Respiratory	−0.63	−1.03	−0.23	0.00
MI	−0.27	−1.27	0.74	0.59
IHD	−0.35	−1.08	0.38	0.35
Cerebrovascular	−0.48	−1.25	0.30	0.23
Arrhythmia	−0.58	−1.37	0.21	0.15
Asthma	−1.60	−2.96	−0.22	0.02
COPD	0.41	−0.62	1.45	0.43
Pneumonia	−0.12	−0.78	0.54	0.72

Table 2. Percentage change in daily hospital admissions for cardiorespiratory diseases (18–64 years age group) associated with 1 °C increase in daily mean temperature during summer months in Helsinki metropolitan area, Finland, 2001–2017.

Outcome	% change	Low CI (%)	Up CI(%)	*p*-value
Cardiovascular	−0.66	−1.33	0.03	0.06
Respiratory	−0.17	−0.92	0.58	0.65
MI	−1.14	−2.91	0.65	0.21
IHD	−0.87	−2.22	0.50	0.21
Cerebrovascular	−0.03	−1.51	1.47	0.97
Arrhythmia	−0.84	−2.46	0.80	0.31
Asthma	0.55	−1.99	3.15	0.67
COPD	1.18	−0.99	3.40	0.29
Pneumonia	−0.54	−1.80	0.73	0.40

Table 3. Percentage change in daily hospital admissions for cardiorespiratory diseases (65–74 years age group) associated with 1°C increase in daily mean temperature during summer months in Helsinki metropolitan area, Finland, 2001–2017.

Outcome	% change	Low CI (%)	Up CI(%)	*p*-value
Cardiovascular	−0.04	−0.79	0.72	0.92
Respiratory	−0.17	−1.12	0.79	0.73
MI	0.51	−1.60	2.67	0.64
IHD	0.02	−1.48	1.54	0.98
Cerebrovascular	−0.29	−1.84	1.29	0.72
Arrhythmia	0.14	−1.56	1.86	0.88
Asthma	−3.86	−7.57	0.01	0.05
COPD	0.40	−1.35	2.19	0.65
Pneumonia	−0.34	−1.86	1.20	0.66

Table 4. Percentage change in daily hospital admissions for cardiorespiratory diseases (≥75 years age group) associated with 1 °C increase in daily mean temperature during summer months in Helsinki metropolitan area, Finland, 2001–2017.

Outcome	% change	Low CI (%)	Up CI(%)	*p*-value
Cardiovascular	−0.56	−1.04	−0.07	0.02
Respiratory	0.08	−0.60	0.77	0.82
MI	−0.08	−1.55	1.42	0.92
IHD	−0.23	−1.28	0.84	0.67
Cerebrovascular	−0.83	−1.95	0.30	0.15
Arrhythmia	−0.85	−1.93	0.24	0.13
Asthma	−1.72	−4.41	1.04	0.22
COPD	−0.05	−1.61	1.54	0.95
Pneumonia	0.43	−0.55	1.41	0.39

File S5: Lag 4

Table 1. Percentage change in daily hospital admissions for cardiorespiratory diseases (All-ages) associated with 1 °C increase in daily mean temperature during summer months in Helsinki metropolitan area, Finland, 2001–2017.

Outcome	% change	Low CI (%)	Up CI(%)	*p*-value
Cardiovascular	−0.39	−0.72	−0.07	0.02
Respiratory	−0.46	−0.83	−0.09	0.01
MI	−0.49	−1.41	0.45	0.30
IHD	−0.71	−1.38	−0.03	0.04
Cerebrovascular	−0.60	−1.35	0.16	0.12
Arrhythmia	−0.84	−1.60	−0.06	0.03
Asthma	−1.28	−2.63	0.09	0.07
COPD	0.49	−0.51	1.50	0.34
Pneumonia	−0.38	−1.02	0.26	0.24

Table 2. Percentage change in daily hospital admissions for cardiorespiratory diseases (18–64 years age group) associated with 1 °C increase in daily mean temperature during summer months in Helsinki metropolitan area, Finland, 2001–2017.

Outcome	% change	Low CI (%)	Up CI(%)	*p*-value
Cardiovascular	−0.24	−0.87	0.40	0.46
Respiratory	−0.03	−0.73	0.67	0.93
MI	−0.86	−2.50	0.80	0.31
IHD	−1.09	−2.34	0.17	0.09
Cerebrovascular	0.23	−1.23	1.70	0.76
Arrhythmia	−0.40	−1.97	1.20	0.62
Asthma	0.74	−1.78	3.31	0.57
COPD	1.39	−0.73	3.56	0.20
Pneumonia	−0.92	−2.14	0.31	0.14

Table 3. Percentage change in daily hospital admissions for cardiorespiratory diseases (65–74 years age group) associated with 1 °C increase in daily mean temperature during summer months in Helsinki metropolitan area, Finland, 2001–2017.

Outcome	% change	Low CI (%)	Up CI(%)	*p*-value
Cardiovascular	−0.25	−0.94	0.44	0.48
Respiratory	−0.24	−1.12	0.65	0.60
MI	−0.23	−2.22	1.79	0.82
IHD	−0.19	−1.59	1.23	0.79
Cerebrovascular	0.04	−1.46	1.57	0.96
Arrhythmia	−0.16	−1.81	1.53	0.86
Asthma	−1.48	−5.19	2.37	0.45
COPD	−0.05	−1.76	1.69	0.95
Pneumonia	−0.27	−1.75	1.23	0.72

Table 4. Percentage change in daily hospital admissions for cardiorespiratory diseases (≥75 years age group) associated with 1 °C increase in daily mean temperature during summer months in Helsinki metropolitan area, Finland, 2001–2017.

Outcome	% change	Low CI (%)	Up CI(%)	*p*-value
Cardiovascular	−0.55	−1.00	−0.11	0.02
Respiratory	0.04	−0.59	0.67	0.91
MI	−0.36	−1.72	1.01	0.60
IHD	−0.72	−1.70	0.26	0.15
Cerebrovascular	−1.38	−2.45	−0.30	0.01
Arrhythmia	−1.45	−2.50	−0.39	0.01
Asthma	−1.73	−4.39	1.00	0.21
COPD	0.39	−1.13	1.94	0.61
Pneumonia	0.11	−0.83	1.05	0.82

File S6: Lag 5

Table 1. Percentage change in daily hospital admissions for cardiorespiratory diseases (All-ages) associated with 1 °C increase in daily mean temperature during summer months in Helsinki metropolitan area, Finland, 2001–2017.

Outcome	% change	Low CI (%)	Up CI(%)	*p*-value
Cardiovascular	−0.39	−0.70	−0.07	0.02
Respiratory	−0.61	−0.97	−0.24	0.00
MI	−0.68	−1.59	0.23	0.14
IHD	−1.09	−1.75	−0.42	0.00
Cerebrovascular	−0.13	−0.86	0.61	0.73
Arrhythmia	−0.77	−1.53	−0.01	0.05
Asthma	−1.81	−3.14	−0.46	0.01
COPD	0.44	−0.55	1.44	0.38
Pneumonia	−0.50	−1.13	0.13	0.12

Table 2. Percentage change in daily hospital admissions for cardiorespiratory diseases (18–64 years age group) associated with 1 °C increase in daily mean temperature during summer months in Helsinki metropolitan area, Finland, 2001–2017.

Outcome	% change	Low CI (%)	Up CI(%)	*p*-value
Cardiovascular	−0.30	−0.92	0.33	0.35
Respiratory	−0.42	−1.10	0.27	0.23
MI	−1.04	−2.67	0.61	0.22
IHD	−1.44	−2.67	−0.19	0.02
Cerebrovascular	0.51	−0.92	1.97	0.49
Arrhythmia	−0.89	−2.46	0.70	0.27
Asthma	−0.54	−3.00	1.99	0.67
COPD	1.27	−0.79	3.37	0.23
Pneumonia	−1.17	−2.37	0.04	0.06

Table 3. Percentage change in daily hospital admissions for cardiorespiratory diseases (65–74 years age group) associated with 1 °C increase in daily mean temperature during summer months in Helsinki metropolitan area, Finland, 2001–2017.

Outcome	% change	Low CI (%)	Up CI(%)	*p*-value
Cardiovascular	−0.01	−0.69	0.67	0.97
Respiratory	−0.03	−0.90	0.85	0.95
MI	−0.61	−2.52	1.33	0.54
IHD	−0.41	−1.78	0.98	0.56
Cerebrovascular	0.54	−0.94	2.05	0.47
Arrhythmia	0.31	−1.31	1.95	0.71
Asthma	−0.95	−4.64	2.89	0.62
COPD	0.11	−1.57	1.82	0.90
Pneumonia	−0.58	−2.04	0.90	0.44

Table 4. Percentage change in daily hospital admissions for cardiorespiratory diseases (≥75 years age group) associated with 1 °C increase in daily mean temperature during summer months in Helsinki metropolitan area, Finland, 2001–2017.

Outcome	% change	Low CI (%)	Up CI(%)	*p*-value
Cardiovascular	−0.60	−1.04	−0.16	0.01
Respiratory	0.00	−0.62	0.62	1.00
MI	−0.53	−1.85	0.81	0.44
IHD	−1.20	−2.16	−0.24	0.01
Cerebrovascular	−0.81	−1.86	0.25	0.13
Arrhythmia	−1.26	−2.29	−0.22	0.02
Asthma	−1.92	−4.52	0.75	0.16
COPD	0.21	−1.30	1.74	0.79
Pneumonia	0.15	−0.77	1.08	0.75
